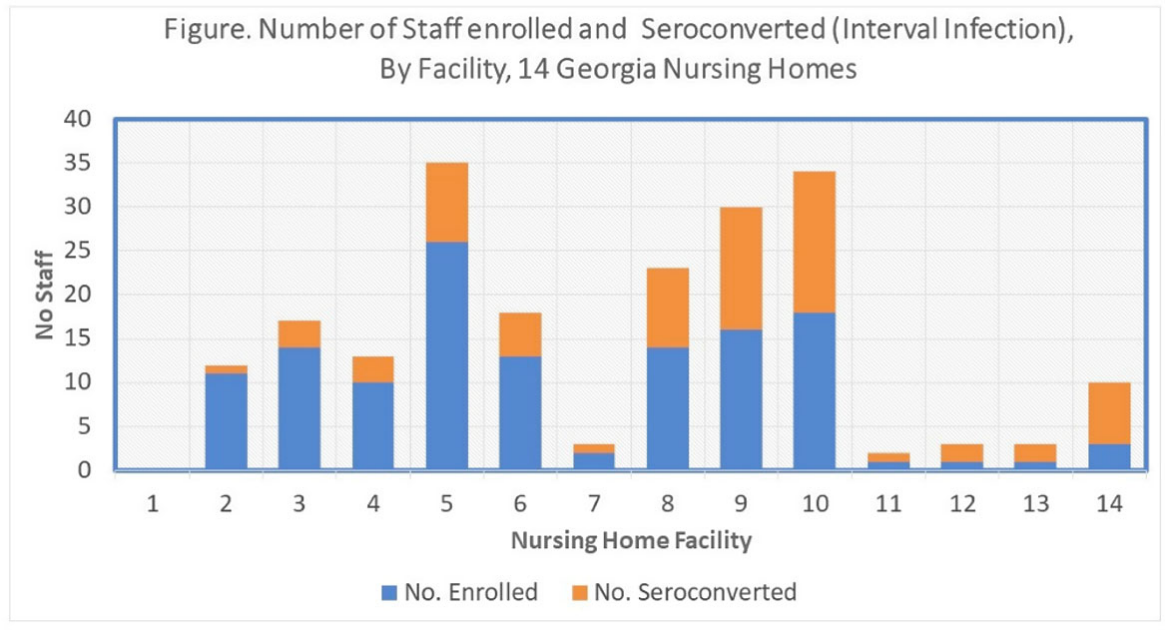# Which nursing home workers were at highest risk for SARS-CoV-2 infection during the November 2020–February 2021 winter surge of COVID-1?

**DOI:** 10.1017/ash.2022.66

**Published:** 2022-05-16

**Authors:** Joseph Kellogg, William Dube, Carly Adams, Matthew Collins, Theodore Lopman, Theodore Johnson, Avnika Amin, Joshua Weitz, Scott Fridkin

## Abstract

**Background:** Nursing home (NH) residents and staff were at high risk for COVID-19 early in the pandemic; several studies estimated seroprevalence of infection in NH staff to be 3-fold higher among CNAs and nurses compared to other staff. Risk mitigation added in Fall 2020 included systematic testing of residents and staff (and furlough if positive) to reduce transmission risk. We estimated risks for SARS-CoV-2 infection among NH staff during the first winter surge before widespread vaccination. **Methods:** Between February and May 2021, voluntary serologic testing was performed on NH staff who were seronegative for SARS-CoV-2 in late Fall 2020 (during a previous serology study at 14 Georgia NHs). An exposure assessment at the second time point covered prior 3 months of job activities, community exposures, and self-reported COVID-19 vaccination, including very recent vaccination (≤4 weeks). Risk factors for seroconversion were estimated by job type using multivariable logistic regression, accounting for interval community-incidence and interval change in resident infections per bed. **Results:** Among 203 eligible staff, 72 (35.5%) had evidence of interval seroconversion (Fig. 1). Among 80 unvaccinated staff, interval infection was significantly higher among CNAs and nurses (aOR, 4.9; 95% CI, 1.4–20.7) than other staff, after adjusting for race and interval community incidence and facility infections. This risk persisted but was attenuated when utilizing the full study cohort including those with very recent vaccination (aOR, 1.8; 95% CI, 0.9–3.7). **Conclusions**: Midway through the first year of the pandemic, NH staff with close or common resident contact continued to be at increased risk for infection despite enhanced infection prevention efforts. Mitigation strategies, prior to vaccination, did not eliminate occupational risk for infection. Vaccine utilization is critical to eliminate occupational risk among frontline healthcare providers.

**Funding:** None

**Disclosures:** None

**Figure:**